# Perspectives and Experiences of Stakeholders on Self-Disclosure of Peers in Mental Health Services

**DOI:** 10.1007/s10597-024-01287-2

**Published:** 2024-05-10

**Authors:** Inbar Adler Ben-Dor, Eran Kraus, Yael Goldfarb, Alina Grayzman, Bernd Puschner, Galia S. Moran

**Affiliations:** 1 The Charlotte B. and Jack J. Spitzer Department of Social Work, Ben GurionUniversity of the Negev, Be’er Sheva, Israel; 2 Department of Psychiatry II, Ulm University, Ulm, Germany; 3 The Academic College of Tel Aviv Yaffo, Tel Aviv-Yaffo, Israel

**Keywords:** Peer support, Peer specialist, Self -disclosure, Use of knowledge from experience /lived experience, Challenges, Recovery oriented organization

## Abstract

With the movement towards recovery-oriented mental health (MH) services, individuals with MH lived-experience are increasingly employed as peer providers (peers). Peers are unique in that they bring knowledge from experience and eye-level connection to service users that enhance the quality of services and humanize MH systems’ culture. In Israel, hundreds of peers are employed in various roles and settings across the MH system. However, peer integration into MH services faces challenges. One issue involves the use of self-disclosure (SD) in MH services which varies with explicitness across roles and settings. This study sought to understand perspectives and experiences regarding peers’ SD (use & sharing of knowledge from experience) among different stakeholders in MH health services. Six focus groups and 4 semi-structured interviews (N = 42) were conducted as a part of a larger international project (UPSIDES; ERC Horizon 2020, Moran et al., Trials 21:371, 2020). Data was transcribed verbatim and analyzed using thematic analysis. Four categories and 7 themes were identified regarding current perspectives and experiences with peers’ SD in MH organizations: (i) Restrained or cautious organizational approach to SD; (ii) Attitudes of peers to SD approach; (iii) The influence of designated peer roles on SD; and (iv) Unwarranted SD of peers working in traditional roles. The findings reveal that peers’ SD in MH services is a complex process. Organizational approaches were often controlling of non-designated peers’ SD practices; participants had diverse attitudes for and against peers’ SD; SD occurred according to personal preferences, specific peer role and the director’s approach to peers’ SD; Conflictual SD dilemmas emerged in relation to service users and staff. SD sometimes occurs unwarrantely due to ill mental health. The presence of peer-designated roles positively impacts peers' SD. We interpret the current mix of views and general conduct of peer SD practice in statutory MH services as related to three aspects: 1. The presence of a traditional therapeutic SD model vs. a peer SD model – with the former currently being dominant. 2. Insufficient proficiency and skill development in peers’ SD. 3. Stigmatic notions about peer SD among service users and staff. Together, these aspects interrelate and sometimes create a negative cycle create tension and confusion.

A need to develop professionalism of peer SD in statutory services is highlighted alongside enhancing staff and service user acknowledgement of the value of peer SD. Developing peer-designated roles can positively impacts peer SD in MH statutory services. Training, support, and organizational interventions are required to further support for peer-oriented SD and the enhancement of a person-centered and recovery orientation of MH services.

## Introduction

Over the last several decades, health-care systems increasingly recognized peer support services as a valuable component in developing recovery-oriented Mental Health (MH) services (Mahlke et al., 2014; Myrick & Del Vecchio, 2016). Peers help service-users by role-modeling that recovery is possible, sharing knowledge from experience and using reciprocal empathic relationships in order to provide hope and support (Davidson & Guy, 2012; Puschner et al., 2019; Repper & Carter, 2011). Peers bring knowledge and skills that are distinct from clinical and academic knowledge that prevails in MH services. Their unique contribution involves ‘having been there’, a sense of camaraderie, a deep understanding, hope giving as well as street wisdom and tips on ‘how to navigate the system’ (Davidson & Guy, 2012; Moran, 2017). These peer ‘essences’ also play a vital role in catalyzing recovery orientation within organizations (Cook et al.,  2012a, b; Mahlke et al., 2014; Moran et al., 2013; Walker & Bryant, 2013).

While carrying a similar essence as described above, peer roles may have differently named titles according to settings, tasks, and organization and involve different means and emphases on sharing one’s lived experience while using self-disclosure (SD). Common peer-role names used are peer support workers, peer specialists, consumer-providers, peer providers, experts by experience, etc. (Rogers et al., 2005; Richard et al., 2009; Solomon, 2004; Slade et al., 2014). For example, peer support can be provided as a group intervention in the community (Bochicchio et al., 2023; Cook et al., 2020; Moran et al., 2020), an individual intervention (Bailie et al., 2016; Truong et al., 2019) for people hospitalized (Hornik‐Lurie et al., 2018; Kivistö et al., 2023; Otte et al., 2020) or as part of multidisciplinary teams in the community (Bailie et al., 2016; Bochicchio et al., 2023; Byrne et al., 2022a; Davis & Pilgrim, 2015; Gillard et al., 2015; Mancini, 2018; Moran et al., 2013). Furthermore, peer support can be offered as an alternative to, an independent service within, or an integral part of professional care (Mahlke et al., 2014; Salzer & Liptzin-Shear, 2002; Slade et al., 2014). Here, we use the term ‘peers’ as a unifying generic description of all individuals employed in peer roles, which involve giving support based on self-disclosure and lived experiences.

## Self-Disclosure in Mental Health Peer Support

Irrespective of role or setting, MH peer support is based directly on the knowledge and learnings from personal experience of facing mental distress (Repper & Watson, 2012). Through SD, peers share their experience with their illness, its psychosocial implications, and their encounter with the MH system (Bailie et al., 2016; Harrington, 2012; Moran et al., 2020; Truong et al., 2019). Peers can offer empathy, validation and establish non-hierarchical, reciprocal, and empowering relationships with service users. By using SD, peers generate trust and closeness, foster hope for recovery, establish themselves as role models, stimulate positive behavioral changes, and promote self-care among service users (Davidson & Guy, 2012; Moran, 2017; Solomon, 2004; Truong et al., 2019).

## Challenges of Peers’ Self-Disclosure in MH Services

Peer support has its roots in the consumer movement and has developed initially outside the mainstream of the MH system (Anthony, 1993; Faulkner, 2017). Peer roles can potentially be a positive disruption to mainstream MH services, challenging the basic assumptions governing MH systems and the way these systems operate (Deegan, 2011). One of these challenges involves the use of SD in mental health services (Chinman et al., 2010; Kemp & Henderson, 2012; Moran et al., 2013; Moran, 2017; Richard et al., 2009; Salzer et al., 2009; Slade et al., 2014). SD-related challenges arise for several reasons: First, sharing one’s personal story, distress, recovery, diagnosis, encounters with the MH system, etc., is a complex task. It requires one to be self-cognizant about the recovery process and well-skilled in sharing parts of one’s personal story with service users, given their needs at different times (Grundman et al., 2021; Moran, 2017). Second, MH ill conditions are often visually “transparent” (Goffman, 2009). This leaves the choice of disclosure (if, what, when, to whom, etc.) at the peers’ discretion. While this may allow peers to consider the pros and cons of SD, such continuous introspective re-evaluation may also be cognitively and emotionally taxing (Brill-Barniv et al., 2017; Mancini & Lawson, 2009). Third, unlike other health conditions, mental illness is still plagued with stigma. Accordingly, ignorance, prejudice, and discrimination are also prevalent in MH services (Corrigan & Rao, 2012; Corrigan et al., 2009; Thornicroft et al., 2007). This means that employing SD might elicit a sense of risk or shame even after becoming proficient at self-disclosure (Bril-Barniv et al., 2017; Mancini & Lawson, 2009). As well as risk the experience of lower status, rejection, and discrimination by other colleagues and/or service users (Corrigan & Rao, 2012; Corrigan et al., 2009; Thornicroft et al., 2007).

## Self-Disclosure in Mental Health Therapeutic Tradition

When employed within statutory MH services, peer SD practices also involves a clash between two different conceptual models for SD. MH services are often based on a traditional therapeutic model of SD. Thus, the definition of SD is different. SD by therapists/MH practitioners may involve revealing personal information regarding the therapeutic process, such as thoughts and feelings, or personal information outside the therapeutic process (Hill & Knox, 2002; Knight, 2012; Henretty & Levitt, 2010). SD in therapeutic models is defined as an occurrence in which a therapist/MH practitioner verbally reveals personal information about themselves in a therapeutic process. Most importantly, It is perceived as a marginal part of care practice that occurs only rarely and needs to be done cautiously (Barnett, 2011; Hill & Knox, 2002; Knight, 2012; Kronner, 2013; Zur, 2007). Historically, under the influence of Freudian psychoanalytic principles, any type of therapist SD was approached with an objection, highlighting its potential negative consequences (Ziv-Beiman et al., 2017). These included the disruption to transference processes within the therapist-client relationship (Archard, 2020), violation and blurring of therapeutic and ethical boundaries (Audet, 2011: Barnett, 2011), and shifting the focus away from the patient (Audet & Everall, 2010; Farber, 2006).

Since the 1950s, humanistic, existentialist, intersubjective, multicultural, and feminist-oriented therapeutic models supported a more positive view of using SD (Henretty & Levitt, 2010; Zur, 2007). Positive consequences of therapist SD were acknowledged as reducing hierarchy and imbalance, promoting therapeutic alliance based on mutual respect, authenticity, cooperation, and confidence; strengthening the authenticity and credibility of the therapist being a role model; and helping to normalize the patient’s experiences (Audet & Everall, 2010; Henretty & Levitt, 2010). Yet, even though SD has become more acceptable within the therapeutic model, it remains the exception rather than the norm. It is controversial, and many MH practitioners avoid using SD or address it cautiously (Henretty & Levitt, 2010; Lovell et al., 2020; Knight, 2012). Within this context, SD of mental illnesses is considered even more controversial (Henretty & Levitt, 2010). Thus, as peer roles continue to emerge within MH statutory services—the sharing of lived experience by peers naturally elicits new questions and concerns about its practice, above and beyond the inherent challenges described in the previous section.

## The Context of the Current Study and Research Question

In Israel, peers have continuously entered the MH formal system over the past 15 years. First, a consumer-provider course was initiated in 2006 by a few individuals with lived experience, which was immediately supported by the governmental MH rehabilitation school (Moran, 2018). Following this, MH service-users were employed in different services in para-clinical roles across the MH system (e.g., rehab instructor, social companion, ADL supporter etc.). For these peer job roles, there were no a priori definite instructions regarding if, when, and how to use SD. Accordingly, we term these para-clinical roles for peers as *non-designated peer roles*. Some of these peers could address the challenges they experience with SD using supervision and support from supported employment services offered to them as consumer-providers. Over time, and as the value of peers’ knowledge was increasingly recognized – designated peer roles have been further developed. In these, lived experience and experiential knowledge were defined as an advantage of job descriptions. These roles included peer specialist in hospitals, peer advocates in the community, UPSIDES peer support group facilitators and more. SD is an explicit job requirement in these roles, key to one’s work role (Lachman et al., 2018; Moran, 2018; Moran et al., 2020; Naaman, 2018). As a result, hundreds of individuals engage in peer roles and face the opportunity to employ self-disclosure on a daily basis, with varying inclinations to use it or share their experiences in different ways.

Studies about the SD of peers in MH services are rare (Truong et al., 2019). This study seeks to understand the experience and attitudes regarding peers' SD of various stakeholders in the Israeli MH system: peers in different roles, non-peer MH providers, and directors of peers in MH staff. We ask: How do peers in different roles handle and view SD within MH services? How do other stakeholders (non-peer MH providers, including staff and directors) experience and view SD?

## Methods

### Research Design

To learn about the experiences and attitudes regarding peers' self-disclosure in the Israeli MH system among various stakeholders (peers, non-peer MH providers, and directors working together in MH staff teams), a qualitative research approach was used involving interviews and focus groups.

#### Setting

The study was part of a larger research project: UPSIDES—Using Peer Support in Developing Empowering Mental Health Services—a 5.5-year (2018–2023) European Union-funded international study. UPSIDES’ goal was to develop and scale up MH peer support internationally, empower MH services, and create an international community across several countries in three continents of peer support workers, professionals, and researchers (Moran et al., 2020; Puschner et al., 2019). To learn about the state of peer support in each country involved in the project, one part of the study involved taking perspectives from different stakeholders in each site about the current state and characteristics of peer support services, described elsewhere (Moran et al., 2020). The current study uses the data from the Israeli site alone in order to investigate the topic of SD, which was uniquely salient in the Israeli site.

#### Participatory Action Research Aspects of the Study

Peers and other relevant stakeholders were involved in various aspects of the project, aspiring for patient and public involvement in all stages of the extensive research project (Bennett & Roberts, 2004; Pinfold et al., 2015). Peers were not just interviewed for the study but also took part in peer training and intervention implementation and were involved in bi-annual meetings of a steering committee for the project. Furthermore, during the analysis phase, the first author presented the initial results to leading peers in the field, who commented on and further influenced the interpretation process.

### Participants

The inclusion criteria were relevant stakeholders with a minimum of 3 months experience, including: a) Directors of peers; b) MH providers working in an MH team with peers. c) Persons working in peer roles in MH services (designated and non-designated) d) Key informants knowledgeable about the implementation of peer support in mental health settings.

Forty-two stakeholders were recruited to the study, including 13 peers in various roles (peers in non-designated roles and peers in various designated roles such as peer specialists in hospitals, peers who participated in the UPSIDES pilot initiative, etc.). 26 MH providers and 3 MH providers with lived experience.

### Peers (n = 13)

Two peers in non-designated roles worked as social support companions and four peers participated in the UPSIDES pilot and, at the same time, worked in traditional para-clinical (non-designated peer) roles in community rehabilitation services (i.e., as social support companions and rehabilitation instructors). Seven worked in peer-designated positions as peer treatment coordinators, peer specialists in hospitals, and professional peer supervisors. Their average age was 37 (28 – 56), 8 were women (62%), 4 (50%) were in a permanent relationship or married, and 4 (50%) were single; for five, there was no information regarding personal status. They had an average of 14.37 years of education (12–17 years) and worked in MH services for.5 5 years on average (1–10 years).

### MH Providers (n = 26)

21 (72%) were social workers, 2 (7%) were occupational therapists, 2 (7%) were organizational consultants, 1 (3%) was an expressive arts therapist, one had a M.A. degree in community mental health (3.5%) and 2 MH providers’ professional background was missing. Fifteen were MH directors of supported community housing, peer programs, and MH professional supervisors. 2 MH providers previously worked in The Ministry of Health in leading positions. The rest (12) were treatment coordinators, rehabilitation counselors, or social support companions' coordinators. All are representatives of various leading organizations in applying peer knowledge from experience in Israel (governmental organizations, consumers run organizations’, NGOs, and private initiatives) All but one had past or actual experience in directing and/or working with peers. The average years working in MH services was 10.86 (2–35). Their average age was 41.17 years (25–63), 18 (62%) were women, 20 (69%) were married, 1 (3.5%) was in a relationship, 5 (17%) were single, 2 (7%) were divorced and 1 (3.5%) was a widow. The average years of education was 18.41 years (17–22).

Three male MH providers had lived experience. 2 were social workers, and 1 had an M.A. in community MH. The average number of years working in MH services was 12.5 years (4–24). Their average age was 44 years (38–49), 2 (67%) were married, and 1 (33%) was divorced. The average number of years of education was 16.3 years (15–17).

Further information about the participants is presented in Table 1 below.

**Table 1:** Participants demographic characteristics

	MH providers		Peers	
N	29		13	
	M (SD)		M (SD)	
Age (years)	41.17 (7.63)		40.5 (9.2)	
Education (years)	18.41 (3.02)		14.38 (2.13)	
Work experience in MH services (years)	10.86 (7.94)		5.5 (2.71)	
	n	%	n	%
Gender				
Women	18	62	9	69
Men	11	38	4	31
Marital status n (%)				
Single	5	17	4	50
Married	20	69	3	38
In relationship	1	4	1	13
Divorced	2	7	-	-
Widow	1	4	-	-
Missing			5	38.5
MH work roles				
Social workers	21	72	-	-
Occupational therapists	2	7	-	-
Organizational consultants	2	7	-	-
M.A. degree in Community mental health	1	4	-	-
Expressive arts therapist	1	4	-	-
missing	2	7	-	-
Peers work roles				
Peers in non-designated roles	-	-	2	15
UPSIDES peers	-	-	4	31
Peers in designated roles	-	-	7	54

^*^3 Male MH providers also had lived experience of mental health problems, their Mean age was 44 ranged from 38–49(SD5.57, two were married and one was divorced. They were highly educated with 15–17 years of education (Mean 16.33, SD 1.15), 2 were social workers and one had a M.A. degree in Community mental health. One was working as a MH provider for 4 years, another for 9.5 years and a third for 24 years

### Procedure

Study participants were selected using purposive sampling (Patton, 1990). The purposive sampling aimed to select stakeholders that were embedded or related to MH work environments where persons with lived experience (peers) were employed to get the perspectives and views of those most directly involved in and with peer MH work in MH services.

Potential participants were contacted in person, by email, or by phone, explaining the study aims and procedures. Those interested in participating received an invitation (via e-mail) with specific dates and venue. Before the focus group sessions and interviews, participants received a verbal explanation of the study and an information sheet about study procedures. Participation was voluntary, and subjects provided written informed consent**.** In the initial stage (between October 2018 to January 2019), four exploratory interviews were conducted with a few key informants in the research field to map the field. Then, six focus groups were conducted. Each lasted 60 to 90 min. Participants received a small multi-purpose shopping voucher worth 80 ILS (approx. 20 dollars) as a token of their participation (Table 2).

**Table 2:** Number of participants in focus groups

Research Population	# of groups	# of participants
MH providers	3	18 (1 with LE*)
MH providers and peers	2	8 Peers 7 MH providers (2 with LE*)
Peers who participated in the UPSIDES pilot	1	4
		24 – Total of MH providers
		12—Total of Peer providers
Total	6	37

### Ethical Considerations

The study was approved by the European Ethics approval and the local University Ethics Committee**.** Codes were used to conceal participants’ identities to ensure confidentiality and anonymity. The rights and dignity of research participants were duly respected and protected by ensuring that their basic human rights, such as the right to privacy and information, among others, were not violated.

### Data Analyses

Using MAXQDA 2020, interview and focus group transcript data were analyzed specifically for the study question and involved reading and re-reading the transcripts for familiarization, coding, and generating themes by the first author. An initial list of codes was developed based on the first three focus group transcripts. This list was discussed with the last writer until a consensus was reached. Based on that list, the remaining transcripts were coded. Coding was conducted using open coding, comparing similarities and differences in the texts, which involved reiterative, inductive, and reductive processes that organized the data (Braun & Clarke, 2006; Walker & Myrick, 2006). New themes that did not fit existing codes resulted in forming new codes, re-conceptualization of previous codes, merging codes, and/or elimination of codes, eventually forming sub-codes, codes, and categories.

### Trustworthiness of Findings

To ensure trustworthiness in this study, Lincoln and Guba's (1985) and Shenton’s (2004) suggestions were observed as follows: During the analysis process, the first and the last author independently analyzed two of the focus groups transcripts and then compared their findings, followed by subsequent discussions to either reach consensus or eliminate non-reconcilable themes (credibility). We present adequate information about the study setting (transferability) and provide a detailed description of the study methodology to allow for repetition (dependability). Finally, data were examined and re-examined during data collection and analysis to guarantee the possibility of replication. Furthermore, the findings were presented and discussed in four conferences and learning forums with peers and non-peer MH providers representing the study sample (Confirmability).

### Findings

We identified four categories (with seven themes) depicting experiences and perspectives of stakeholders on peer SD in MH services: (i) Restrained or cautious organizational approach to SD; (ii) Attitudes of peers; (iii) Influences of designated peer roles; (iv) and Instances of unexpected SD (see Table 3).

**Table 3:** Self-disclosure of peers in MH services: categories and themes

Category	Themes
1. Restrained or cautious organizational approach to SD	a. Controlling SD is helpful for both service users and peers b. Fear of stigma c. Respect for peer's freedom of choice
2. Attitudes of peers to SD approach	a. Peers who oppose a restrained and cautious organizational approach b. Peers who adhere to the restrained approach
3. The influence of designated peer roles on SD	
4. Unwarranted SD of peers working in traditional roles	c. Uncoordinated SD leads to rupture with service user and/or staff d. Forced SD due to unexpected ill mental health

1. Restrained or cautious organizational approach to SD

Study participants noted that organizations often approached peers’ SD in a restrained or cautious manner. Directors instructed peers (in non-designated roles and others working both in designated and non-designated peer roles) to avoid sharing their lived experience as default. One peer participant (1) said: "Many directors do not encourage disclosure. As a matter of policy in most organizations, disclosure is not encouraged".

Another said:

[…] "When you enter a housing service [in a non-designated peer role], it’s a little more complicated because you are under the collective decision of the organization. They will accept self-disclosing to staff, but they will not accept self-disclosing to residents. And it happens a lot". Peer in designated role (2).

Overall, this restrained approach is endorsed by participants for three main reasons described next:

- Controlling SD is helpful for both service users and peers

Not using SD as default was appreciated and framed as a carefully well-thought-out process and the proper thing to do. For example, a non-peer MH provider said (3):

[...]"In the first encounter between a social supporter [a non-designated peer role] and a service user...I always tell the social supporter – you will need to say a few things about yourself, but under no circumstances should you self-disclose in the first meeting... because I believe the process of disclosure should be very clear and precise, and it should take place after the establishment of an initial relationship, and we need to consider... whether it will contribute to the relationship or not."

Another non-peer MH provider (4) said:

"[…] I always tell them [i.e., peers in a non-designated role]: ask yourselves if this is meant to do something for you or for the resident] service user using a residential service, I.A.B.]? If you self-disclose when it doesn’t serve the resident but only serves you, it hurts the resident and your relationship. ...that’s very important for me."

Overall, non-peer MH providers and some peers often encouraged disclosure only after careful preparation and coordinating expectations between MH providers and peers in non-designated roles. SD was considered beneficial to service users only after establishing contact and becoming familiar with service users.

b. Fear of stigma

Another reason for supporting a cautious approach to SD was the fear of stigma that might arise among MH providers and service users. As a non-peer MH provider (13) explained:

"I’ve been working in this organization for five years now, … and I prefer that consumer-providers [peers in non-designated role, I.A.B.] will not [disclose] because from my experience, social workers and rehabilitation workers also have [stigma]".

A peer in a non-designated role (5) added:

"I, for example, am not exposed in our service. We ourselves know – among the colleagues who are consumers-providers and don't tell the rest of the staff. There is a stigma and lack of knowledge among staff. Many times, the staff refers to service users as “they,” and we sit and exchange looks with each other [meaning that] we are like you, we are the same, not "they or them."

In addition, there is a concern that service users will not be interested in liaising with a service-provider who has lived experience. As one non-peer MH provider (6) said:

"[…] There are, say, service users, who will not accept or even kick out a rehabilitation instructor if they find out that they’re a consumer-provider... such things have already happened... for some service users it might fit perfectly, and for others, it might be seen as an insult to their ego – "Why are you sending me someone lesser?".

Another non-peer MH provider (4) adds: "[…] The self-stigma that the residents have – it’s amazing how they all say it eventually – it’s like, “I deserve something better; I deserve someone normal."

c. Respect for peer’s freedom of choice

A third explanation in support of current organizational approaches addressed peers’ right to self-disclose only when it feels appropriate to them:

"[…] I think that as rehabilitation workers [non-designated peer role, I.A.B.], we have many other experiences as well… whether it’s a physical illness or anything like that, and we won’t always disclose that. So, I also don’t think that all consumer-providers must self-disclose, it’s their business. And yes, it does involve the idea that we’re giving them freedom of occupation[…]"—non- peer MH provider (7).

A non-peer senior policymaker (8) also addressed this issue: "The price of exposure can be high. You must be careful not to push people to self-disclosure… this is a person's choice, and in such a choice, there can be changes […]."

Overall, participants point to a conservative peer SD approach reflected in policies and directors’ approaches, justified due to issues of service-user considerations, stigma, and freedom of choice in peer SD.

2. Attitudes of peers toward the SD approach

Peers in the study did not have a uniform perspective toward the SD approach practices customary in MH organizations. While most of them were opposed to the cautious approach and experienced it as limiting their freedom to share lived experience and self-disclose their MH condition. Some peers did view this organizational norm as justified and appropriate.

- Peers who oppose a restrained and cautious organizational approach

Most peers perceived the organizational norms towards peer SD as externally dictated and potentially harmful. One peer in a non-designated role (2) said:

"The organization can decide and explain it [why not to disclose, I.A.B.], and then you do what the organization asks ... in the meantime, many mistakes are made in your employment. There are many places in which you can use your knowledge from experience, whether directly or indirectly, and these options are closed-off to you because the organization thinks it’s not right for us, for service users, for the staff, whatever".

A peer in a designated role (9) further shared her frustration about when she worked in a non-designated peer role:

"The fact that a case-manager can tell you – you can’t self-disclose to this person, you can self-disclose to that person [...] It’s an issue. […] Someone with knowledge from experience, it’s something that is completely personal that you are being asked to erase. They’re basically telling me to erase a part of myself... A part that can be very easily found out[...]"

b. Peers who adhere to the restrained approach

However, some peers agreed with the existing approach and claimed it still leaves room for them to have a choice on the matter. For example, a peer in a designated role said:

"I learned that my knowledge from experience is divided into stories: There is a specific story for each situation, it is not that a service user comes, and you spill it out [i.e., your personal story, I.A.B]. This is the right way. You must think about where, how much, and why you self-disclose". (1).

Another peer in a designated role (10) said:

"I work with two [ service users, I.A.B.] that I am not exposed to because it is not an issue there – there are no conversations about life and life experience [ ... [on the other hand, anyone I converse with, I [usually] self-disclose to [them]. So, I don't have many dilemmas".

3. The influence of designated peer roles on SD

Participants noted that the introduction of designated peer roles created tensions and opportunities for peers’ SD practices. Participants mentioned that when they had opportunities to engage in designated peer roles (such as UPSIDES group facilitator), they felt more comfortable to self-disclose, and the stigma around lived experience decreased. For example, a peer (12) employed in the past in a non-designated peer role, who also became involved in the UPSIDES group (a peer-designated role) said: "This pilot [UPSIDES, I.A.B.] actually gave me an opportunity to reconsider the possibility to totally self-disclose".

Likewise, a long-time peer in a designated role (11) described the impact she sees on the insertion of designated peer roles as follows:

"With the creation of peer specialist positions, there is greater legitimacy to self-disclose and use knowledge from experience[...] Today there are people who work in the position of peer specialist, who are proud of their work and walk with their heads held high. In the past, there was the sense that a person with knowledge from experience needed to "come out of the closet." Today, as well, there’s a lot of stigmas around the matter, but we’ve made significant progress with creating the peer specialist position".

On the contrary, one non-peer MH provider (8) with senior experience was more reluctant about the push for peer SD through peer-designated roles, claiming that:

"Nowadays, the entire policy is aimed at disclosure. Systems want to show that we have so and so workers [with mental health lived experience, I.A.B.]. You can only receive government support/matching if you employ self-disclosed people. …. Still, the stigma remains considerable. People who self-disclose pay a significant personal price".

4. Unwarranted SD of peers working in traditional roles

Despite the desire to control peers’ SD process, several participants noted that unplanned disclosure sometimes occurs despite coordination between peers in non-designated roles and MH directors/staff. In addition, some reported incidents of uncontrolled SD in states of ill MH. Both these instances elicit negative emotions and detrimental effects on peers' relationships with service users and MH providers.

- Uncoordinated SD leads to rupture with service users and/or staff

A non-peer MH Provider (4) who coordinates peers’ work said:

"It happens often... that things blow up. For example, in an initial meeting with a peer [in a non-designated role], we talked about his (lived) experience. I explained that the approach the organization and I take is not to disclose in the first meeting but only to disclose if it serves a purpose. And then in the first meeting, he disclosed his life story to her [i.e., the service user, I.A.B], and what happened then is that the whole thing blew up, and she refused to continue receiving services by him".

Similarly, another non-peer MH provider (6) frustratingly stressed the importance of coordinating peers’ SD with her:

"[… [I know that for this tenant [i.e., service user, I.A.B.], it’s not good that now it's [i.e., SD, I.A.B.] pulled out. But he [i.e., the consumer provider, I.A.B.] doesn't know; he does it innocently because he flows with the conversation. But we agreed in advance not [to disclose] at this moment. Now, even if you want to change the agreement, it's o.k., but again, the coordination issue with the case manager is significant. It's not like if I feel like pulling it[SD, I.A.B.] out – so I pull it out ".

b. Forced SD due to unexpected ill mental health

Another more extreme form of unwarranted SD was instances of forced SD that occurred in states of deterioration of peers’ mental health conditions. For example, a peer (12) shared:

"There was a case when I was hospitalized [due to mental ill-health, I.A.B.] [...] one of my residents, with whom I had good relations – would call to ask how I was doing, and one day she saw that I wasn’t answering, so she called my ex-husband, and he stupidly told her that I was hospitalized. So she was very stressed. ...I encountered very decisive resistance from the social workers who said that due to the disclosure, the resident went into a crisis herself. I said she could’ve heard that I have cancer or was going through an asthma attack, and that would’ve made her anxious, too. She [The social worker, I.A.B.] wanted to fire me, and then I calmed her down, and she agreed not to give up on me".

Notably, this peer was known to the service users from their role as a rehabilitation instructor (a non-designated paraclinical role) and not from being a UPSIDES peer (a peer-designated role).

Thus, some MH providers found uncoordinated SD as harmful, especially when it occurred due to ill MH. Both events were perceived as harming relations with service users or non-peer staff.

## Discussion

Peer roles increasingly develop in statutory MH services (Davidson & Guy, 2012; Puschner et al., 2019). This study sought to understand the experiences and views of different stakeholders about peers SD working in various roles within the Israeli MH system (i.e., both peers in non-designated and designated peer roles). This study question developed in the context of a more extensive study (UPSIDES; Moran et al., 2020) as it caught the authors’ attention due to the intense conversations held by study participants regarding peers’ SD in Israeli MH services. SD emerged in these discussions as a complex challenge entailing diverse experiences and attitudes on behalf of the stakeholders involved.

We interpret the findings addressing three dimensions: *conceptual models of SD, professionalism of peer roles,* and *stigma related to SD in MH.* We gather that elucidating each separately will allow an in-depth understanding of their respective contribution to the current tension and confusion about issues of peers’ SD. These perspectives can be relevant to other researchers, practitioners, and peers interested in implementing peers in MH statutory services.

1. Conceptual models of SD: therapeutic model vs. peer model

Most study participants viewed existing organizational policies as geared to cautious or restrained peers’ SD, especially in non-designated peer roles in MH services. We interpret the adherence to a more controlling approach to peers’ SD as related to the classic therapeutic model of SD, which is dominant in the culture of the MH system. In this model, practitioners do not see sharing personal knowledge as helpful or relevant to their role as providers (Lovell et al., 2020: King et al., 2020; Kottsieper& Kundra, 2017). They use self-disclosing practices (personal and self-revealing acts) cautiously and often will avoid them all altogether (Henretty & Levitt, 2010; Lovell et al., 2020; Knight, 2012).

However, some peer participants in the study advocated a peer model for SD. They were frustrated about the current trend, feeling they couldn’t fully exploit their lived experience for the sake of service users. This echoes other studies showing that peers' SD creates trust and closeness, validates the experiences of service users, fosters hope for recovery, and establishes peers as role models stimulating positive behavioral changes (Davidson & Guy, 2012; Gillard & Holley, 2014; Grundman et al., 2021; Moran, 2017; Solomon, 2004; Truong et al., 2019).

**Fig. 1:**
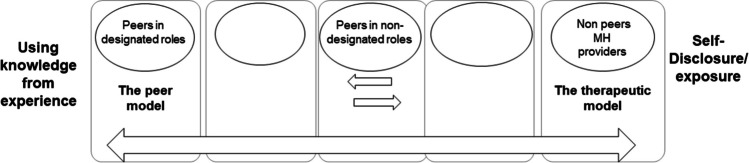
Proximity to the use of knowledge from experience and SD: The therapeutic model versus the peer model

These findings demonstrate two opposite poles representing the therapeutic and the peer models for SD (see Fig. 1). This can be traced in the contrasting linguistic terms used by participants, which signify each model. For example, the therapeutic model employs terms such as "self-disclosure" and "exposure" (Hill & Knox, 2002; Knight, 2012; Henretty & Levitt, 2010). while the peer model addresses SD as " knowledge from experience", "experiential knowledge", "lived experience", and more recently "expertise by experience" (Kivistö et al., 2023). Our finding suggests that participants in this study currently experience a mix of these languages which are confusing without a distinction of their meaning and origins. Participants are affected and make decisions about SD as they rotate between these opposing poles according to each one’s organizational context, the current definition of their roles (non-designated roles or peer specialist roles), MH practitioners’ demands, and personal preferences.

Overall, the current study shows that peers in non-designated paraclinical roles tended to subside to the classic therapeutic SD model, while peers in designated roles were more often led by the peer SD model and more positively acknowledged by their colleagues. The presence of both these roles within the MH system without proper acknowledgment of them and their differences creates confusion: on the one hand, there is recognition of the value of the lived experience for MH services, while at the same time, the therapeutic model of SD is considered the primary instructive guidance for peers’ SD. We believe this confusion explains the extended discussions about the topic among study participants in the Israeli study site of the UPSIDES project (Moran et al., 2020). Role confusion is an element that notably appears in multiple studies as a barrier to implementing peer roles within MH systems (Adams et al., 2023; Ibrahim et al., 2020; Mutschler et al., 2022).

2. Professionalism of peer roles

The study findings reveal additional concerns regarding proficiency in employing peers’ SD. These concerns were exacerbated when SD involved sharing MH challenges, especially when a peer’s MH condition deteriorated and unwarranted SD occurred due to it. Such concerns were identified as unwarranted also with service users in general (Bril-Barniv et al., 2017), even more so when individuals with SMI engage in peer roles (Moran et al., 2013).

Stakeholders related to the need to use SD with preparation and skill. Especially among peers in non-designated roles who did not necessarily get training on SD. Currently, disclosure of mental health difficulties in the workplace is still a complex process with few benefits and many potential repercussions for healthcare professionals not in designated peer roles (Hudson et al., 2021). Therefore, in order to support peer-roles, recognition of the value of lived experience and educating the workforce about mental health difficulties will help work environments become safer for disclosure.

In addition, comprehensive training on the use of SD can meaningfully contribute to peers in non-designated peer roles as well as peer specialists in statutory MH systems. Initiatives are emerging with specific designated training, supervision, and support to develop peer SD skills (Hadas Grundman et al., 2021; Moran et al., 2020). Furthermore, education for MH providers on issues related to lived experience and peer integration into the MH workforce can enhance their readiness to work alongside peers (Byrne et al., 2022b; Kraus & Moran, 2019; Ibrahim et al., 2020).

Training and supervision go beyond gaining skills, especially in traditional services. Often, in these services, there is a clear division between "the healthy practitioner” and the "sick" service user and "we"– the "healthy" MH providers (King et al., 2020). As such, peers are more challenged to integrate different parts of their identity—"service user" and "service provider"—into a single, complex hybrid identity. Gaining proficiency in using lived experience, alongside acknowledgment from MH staff and directors about the value of peer knowledge, could help with participants' current frustration and inability to fully self-express their lived experience and knowledge, which they thought was significant for their role as helpers. At the same time, securing the choice to self-disclose according to one’s level of convenience and respect for their need for freedom in decisions related to SD should be part of professional consideration given the complex contexts and non-designated roles that exist in the system.

- 3. Stigma related to SD in mental health

The findings show that in addition to contrasting models of disclosure (therapeutic vs. peer) and skill development in SD, stigma is still prevalent in participants’ views and experiences of peer SD. For example, director’s restrictions on peer SD that intend to shield service users’ from knowing about the peers’ MH condition, and service users’ that contended that a person with lived experience as a provider will be "lesser" than MH provider. We understand these remarks as evidence of ignorance, prejudice, and discrimination (Thornicroft et al., 2007). Notably, negative opinions of staff or directors about SD, when no one in the organization talks about SD openly, creates the illusion that the team is made up of only “healthy” practitioners and the cultural norm of non-disclosure becomes the norm (Boyd et al., 2016; King et al., 2020; Zerubavel & Wright, 2012). The perception that professionals must be free of any impairment and not allowed to show vulnerability leads to pressure, not to SD (King et al., 2020). Thus, a culture of non-disclosure reinforces the dichotomy between" healthy" MH providers and "sick" service users (Boyd et al., 2016; Harris et al., 2016; Harris et al., 2019). Organizational cultures of mental health services are key in influencing the practice and normalization of SD (Boyd et al., 2016; King et al., 2020; Marino et al., 2016; Morgan & Lawson, 2015), which in turn contributes to the erosion of prevalent stigma.

## Summary and Practical Implications: Peers Facilitating SD Culture as a Compass for Enhancing Recovery-Orientation in Mental Health

The current study examined the experiences and attitudes of stakeholders regarding self-disclosure (using experiential knowledge) among peers (peers in non-designated roles and peers in designated roles) in statutory MH services. Participants voiced complex and sometimes contrasting views and experiences regarding peers’ SD. These diverse and sometimes clashing views are understood when contextualized historically, given the insertion of consumer-providers employees almost 20 years ago into the MH system, followed by additional insertion of various designated peer roles over time (peer specialists, etc.). This resulted in a system where hundreds of individuals with lived experience are engaged in diverse (mostly para-clinical) peer roles.

As peer roles developed in Israel, friction and tension naturally arose against the existing traditional SD therapeutic approach. The mere presence of peers and peer-roles in services appears to challenge the surrounding staff and services users, on traditional assumptions regarding SD practices. This disruption of organizational and cultural norms is not apprehended but rather welcomed as a natural in light of change processes (Deegan, 2011). Peer-designated roles are important by definition because they require the use of SD according to the peer model (Harrington, 2012; Mead et al., 2001; Moran, 2018; Slade et al., 2014; Solomon, 2004; Truong et al., 2019). Such designated peer roles carry unique benefits to service recipients as well as impact MH practitioners who work alongside peers to feel more comfortable to share their own psychiatric histories in their MH service (Byrne et al., 2022a; Harrington, 2012; Weerman & Abma, 2019). This study identified how currently integrating knowledge from experience within MH systems interacts with conceptual models of SD, the issue of professionalism of peers, and the ensuing problem of stigma. We suggest providing organizational support that will encourage expressing one’s lived experience without fear of judgment regarding one’s professionalism (King et al., 2020; Marino et al., 2016; Morgan & Lawson, 2015). In addition, training, supervision, and guidance for staff members and organizations that will address the SD of peer and non-peer employees can raise awareness of the benefits of knowledge from experience and help the proficiency of peers’ SD (King et al., 2020). We see these as essential next steps in developing recovery-oriented MH structures that can contain peer models of SD alongside the traditional therapeutic professional models (Moran, 2017; Dunlop et al., 2022; Harrington, 2012; Moran et al., 2013; Truong et al., 2019).

## Data Availability

Data is available upon request from Inbar Adler-Ben dor email: adlerben@post.bgu.ac.il.
